# Isolated pulmonary valve endocarditis

**DOI:** 10.1007/s00508-024-02416-3

**Published:** 2024-08-23

**Authors:** Sonja Valsky, David Mutschlechner, Dominik Wiedemann, Thomas Gremmel

**Affiliations:** 1 Department of Internal Medicine I, Cardiology and Intensive Care Medicine, Landesklinikum Mistelbach-Gänserndorf, Liechtensteinstraße 67, 2130 Mistelbach, Austria; 2 Karl Landsteiner Society, Institute of Cardiovascular Pharmacotherapy and Interventional Cardiology, St. Pölten, Austria; 3 Karl Landsteiner University of Health Sciences, Krems, Austria; 4 Department of Cardiac Surgery, Universitätsklinikum St. Pölten, St. Pölten, Austria

**Keywords:** Endocarditis, Isolated pulmonary valve endocarditis, Echocardiography, Transesophageal echocardiography, Surgical valve replacement

## Abstract

Isolated pulmonary valve endocarditis (IPE) is a rare form of infectious endocarditis. This article reports the case of a 49-year-old patient with IPE who was initially admitted with suspected cholecystitis. After vegetations were detected by transthoracic (TTE) and transesophageal echocardiography (TEE), antibiotic therapy in accordance with the antibiogram was primarily attempted; however, due to persistently elevated infection parameters and structural valve damage a pulmonary valve replacement was eventually performed.

**Video online:**

The online version of this article contains 3 videos. The article and the videos are available online (10.1007/s00508-024-02416-3). The videos can be found in the article back matter as “Electronic Supplementary Material”.

## Introduction

Isolated pulmonary valve endocarditis (IPE) is a very rare disease with a reported incidence of only 1–2% of all cases of infective endocarditis [1, 2]. Published risk factors include congenital cardiac malformation, right heart failure, sepsis, intravenous drug use, and placement of a central venous catheter or pacemaker [3, 4]. This article describes the case of a 49-year-old IPE patient with systemic bacteremia.

## Case description

**Fig. 1:**
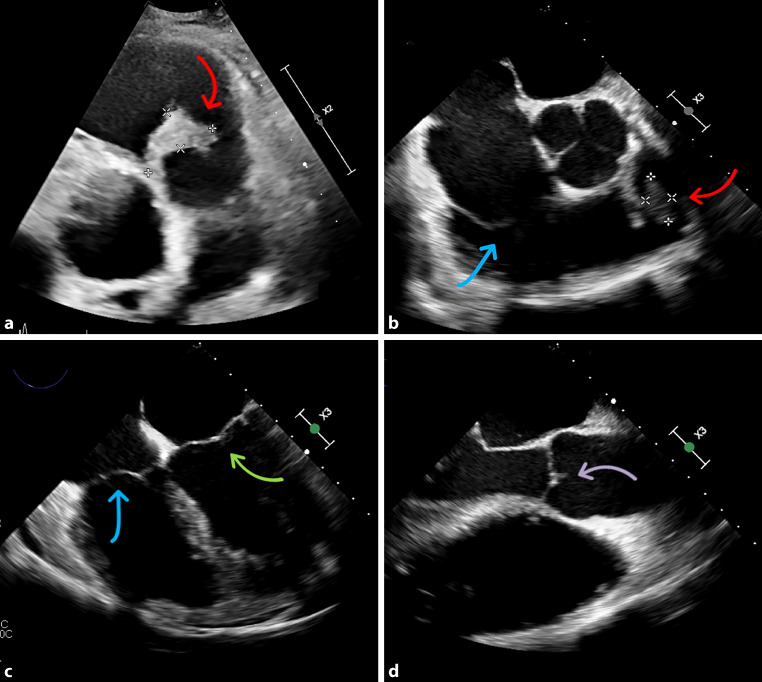
Vegetation at the pulmonary valve in transthoracic and transesophageal echocardiography. **a** Parasternal short axis view showing the vegetation on the pulmonary valve (*red arrow*). **b** Transesophageal echocardiography showing the vegetation at the pulmonary valve (*red arrow*) and the tricuspid valve (*blue arrow*) without any vegetation. **c** Transesophageal echocardiography showing the mitral valve (*green arrow*) and tricuspid valve (*blue arrow*) without any vegetation. **d** Transesophageal echocardiography showing the aortic valve without any vegetation (*purple arrow*)

A 49-year-old male patient presented to the emergency department with fever and body aches. Laboratory tests showed increased infection and cholestasis parameters. Sonography revealed cholecystolithiasis, so the patient was admitted to the gastroenterology ward with suspected cholecystitis. An antibiotic regimen was established, but no improvement in the general condition of the patient could be achieved. Computed tomography of the thorax and abdomen revealed pericardial effusion and multiple peripheral pulmonary emboli. Thus, we performed transthoracic (TTE) and transesophageal echocardiography (TEE), where an isolated pulmonary valve endocarditis with a vegetation of 4 cm was detected (Fig. 1; Videos S1–S3). In the microbiological processing of the blood cultures, *Streptococcus agalactiae, Staphylococcus haemolyticus* and *Staphylococcus pettenkoferi* were detected. The patient was transferred to the cardiology ward and switched to antibiotic therapy with cefazolin and daptomycin in accordance with the antibiogram. Further diagnostic work-up revealed a carious tooth status and a necrotizing ulcer of the right second toe, most likely due to type 2 diabetes. One tooth was extracted and the ulcerated toe was amputated. Moreover, guideline-recommended antidiabetic therapy was initiated. In consultation with the Department of Cardiothoracic Surgery of our partner hospital, a conservative approach was primarily recommended; however, pulmonary valve replacement with a homograft was eventually performed due to persistently elevated infection parameters and the lack of improvement in echocardiographic findings. In fact, subsequent follow-up TEE, as demonstrated in the attached loops S1–S3, revealed progressive valve destruction with subsequent valve dysfunction.

Surgical valve replacement was performed 3 days after the transfer to our partner hospital. A cryopreserved homograft, size 29 mm, (Lifenet Health, Virginia Beach, VA, USA) was implanted. As per standard operating procedure in cases of valve replacement due to endocarditis, cultures were obtained from the valve material; however, microbiological analysis revealed no pathogens on the native valve itself.

Following a 1-day stay in the intensive care unit, the patient was successfully transferred to the cardiothoracic surgery general ward. After a further 8‑day stay, the patient was discharged from inpatient care. The antibiotic therapy, guided by the antibiogram, was administered for 6 weeks.

## Comment

IPE is a very rare variant of infective endocarditis, accounting for only 1–2% of all cases of endocarditis [6]. Its low incidence may be due to anatomical or hemodynamic properties of the pulmonary valve. Documented cases indicate that IPE occurs more often in patients with elevated right ventricular pressure, septic conditions, or intravenous drug use, suggesting a predisposing influence [7, 8]. The question why the pulmonary valve was specifically affected in our patient is more complex. In this case, the bacteremia may have been the result of a recent tooth extraction or, more likely, the consequence of a chronic necrotizing ulcer of the right second toe due to type 2 diabetes mellitus. The blood cultures identified *Streptococcus agalactiae, Staphylococcus haemolyticus*, and *Staphylococcus pettenkoferi*, all of which are recognized causes of bacterial endocarditis, particularly in the presence of predisposing factors such as dental and skin infections. Yet, the microbiological analysis of the native valve material did not reveal any evidence of pathogens; however, this is frequently seen in patients with endocarditis [5]. A similar case in the literature describes an individual with diabetes mellitus and subsequent sepsis, who was diagnosed with pulmonary valve endocarditis [9] underscoring the significance of systemic infections and pre-existing risk factors in the development of IPE.

The need for surgery in IPE depends on various factors, including severity of the infection, occurrence of complications, response to antibiotic therapy, valve function and the overall clinical status of the patient. In some cases, conservative management with antibiotics alone may be successful, particularly in patients with uncomplicated IPE and a favorable response to medical treatment [6]; however, in cases of significant valve destruction with subsequent valve dysfunction, persistent infection despite appropriate antibiotic therapy, development of complications, such as abscess formation or septic embolization, and failure to achieve clinical improvement, surgical intervention becomes necessary [1, 6, 10]. Empirical evidence has demonstrated that surgical measures, such as valve replacement or repair, are frequently indispensable in cases of IPE to effectively eradicate the infection, mitigate the risk of embolic events, and restore cardiac function [11, 12]. The decision to proceed with surgery is typically made on a case-by-case basis, considering the individual patient’s clinical presentation, imaging findings, and response to medical treatment. In our case, the decision for surgical treatment was made primarily due to the unmanageable sepsis and further valve destruction with subsequent dysfunction in the follow-up TEE. The attending cardiac surgeons opted for a homograft for the following reasons: first, homografts in pulmonary position show excellent hemodynamic results. Second, taking into account the young age of the patient (49 years), a homograft was a good choice in terms of durability. Third, from an infectiology standpoint it was appealing to opt for a homograft in order to reduce the amount of xenogenous material. Finally, a homograft was available in a timely manner.

In conclusion, while conservative management may be successful in some cases of IPE, surgical intervention is often necessary, particularly in cases of severe infection or complications. Further research is needed to refine treatment strategies and improve outcomes for patients with this rare but serious condition.

## Supplementary Information

**Video S1: **Transthoracic parasternal short axis view showing the vegetation on the pulmonary valve.

**Video S2: **Transthoracic parasternal short axis view showing the vegetation on the pulmonary valve.

**Video S3: **Transesophageal echocardiography showing the vegetation on the pulmonary valve and the tricuspid valve without any vegetation.

## Data Availability

No new data were generated or analyzed in support of this research.
